# Interleukin-23 receptor signaling mediates cancer dormancy and radioresistance in human esophageal squamous carcinoma cells via the Wnt/Notch pathway

**DOI:** 10.1007/s00109-018-1724-8

**Published:** 2018-11-27

**Authors:** Yuepeng Zhou, Yuting Su, Haitao Zhu, Xuefeng Wang, Xiaoqin Li, Chunhua Dai, Chengcheng Xu, Tingting Zheng, Chaoming Mao, Deyu Chen

**Affiliations:** 1 Institute of Oncology, Affiliated Hospital of Jiangsu University, Jiefang Road 438, Zhenjiang, 212001 China; 2 Department of Medical Imaging, Affiliated Hospital of Jiangsu University, Zhenjiang, 212001 China; 3 Department of Nuclear Medicine, Affiliated Hospital of Jiangsu University, Zhenjiang, 212001 China

**Keywords:** Interleukin-23, M1 macrophage, Wnt/notch, Radioresistance, Biomarker

## Abstract

**Abstract:**

In the tumor microenvironment, inflammatory cells and molecules influence almost every process; among them, interleukin-23 (IL-23) is a pro-inflammatory molecule that exhibits pro- or anti-tumor properties, but both activities remain poorly understood. In this study, we investigated the effect of extracellular IL-23 in IL-23 receptor-positive (IL-23R^+^) esophageal squamous cell carcinoma (ESCC) and explored the mechanisms underlying this effect. We analyzed ESCC tumor tissues by immunohistochemical and immunofluorescence staining and found that IL-23, which was highly expressed, co-localized with Oct-4A in IL-23R^+^ ESCC cells. In addition, IL-23 treatment significantly increased the accumulation of CD133^+^ cells and activated the Wnt and Notch signaling pathways in CD133^−^IL-23R^+^ ESCC cell lines. Consistently, CD133^−^IL-23R^+^ cells pretreated with IL-23 showed stronger anti-apoptosis activity when exposed to radiation and higher survival than untreated groups. Moreover, the inhibition of Wnt/Notch signaling by a small-molecule inhibitor or siRNA abolished the effect of IL-23-induced dormancy and consequent radioresistance. Taken together, these results suggested that IL-23 facilitates radioresistance in ESCC by activating Wnt/Notch-mediated G0/1 phase arrest, and attenuating these detrimental changes by blocking the formation of dormancy may prove to be an effective pretreatment for radiotherapy.

**Key messages:**

- IL-23/IL-23R is correlated with the acquisition of stem-like potential in ESCC.
- CD133^−^IL-23R^+^ ESCCs acquired dormancy via IL-23.
- Radioresistance depends on IL-23-mediated Wnt/Notch pathway activation in vitro and vivo.

**Electronic supplementary material:**

The online version of this article (10.1007/s00109-018-1724-8) contains supplementary material, which is available to authorized users.

## Introduction

Diagnosing esophageal squamous cell carcinoma (ESCC) in the early stage is difficult because the clinical manifestations, such as esophageal obstruction and burning or aching with a concurrent dragging sensation, tend to be ignored or even misdiagnosed [1]. Previous reports showed that ESCC ranks as the sixth-leading cause of cancer death worldwide and its top morbidity and mortality all fasten on China [2, 3]. In fact, most patients die of recurrences after decades of remission due to resistance to current therapies and distant organ metastases. Irrespective of the resectability of lesions, radiotherapy is the optimal choice and first-line treatment for this disease, despite its limited curative effect in cases with resistance [4]. Therefore, determining the critical factors and conditions that affect the formation of radioresistance is the primary step in the development of targeted ESCC therapy.

According to the studies, tumors contain a subset of cancer cells that establish colonization from a non-dividing state of dormancy and ultimately become resistant to therapy [5–7]. Cancer cells with stem cell–like features like dormancy have been identified in hematological diseases and solid tumors widely [8, 9]. However, a better understanding of their mechanism of resistance is needed to isolate, identify, and eliminate these cells, and dissect the responsive signaling pathways. As a matter of fact, cancer cells with stem-like properties are not easily detected because the dormant cell population is often small and thought to be maintained by the resident microenvironment such as stromal cells, secreted growth factors, and local immunoregulation factors [10, 11]. Despite the limited knowledge, cancer cells with stem-like properties are generally accepted to express markers typical of normal stem cells, such as c-myc, Oct-4A, CD133, and CD44 [5]. Although CD133 is the most commonly used marker to isolate cancer stem-like cells in several carcinomas, markers, such as those of therapy resistance, differ by cancer type [5, 8, 11–13]. Likewise, rather than focusing on the identification of optimal markers in specific tumors, research should concentrate on the acquisition of quiescence, self-renewal, or other stemness, and the expression or upregulation of markers constitutes only one method to identify them. Our previous study showed that IL-23 initiates the epithelial–mesenchymal transition (EMT) in ESCC, which allows cancer cells to become motile and invasive [14]. Accumulating evidence shows that cancer cells possessing stem-like properties exhibit an EMT phenotype that enables cells to survive and actively cause relapse, thus a better understanding of the causes of EMT could provide a foundation for tumor treatment [15, 16]. Specifically, tumor-infiltrating lymphocytes (TILs) and their associated cytokines, which are generally associated with better clinical outcomes, play an immune-editing role in cancer, particularly in therapy resistance [10, 17, 18]. However, the tumor-promoting role of immune factors in the formation of cancer stem-like cells has rarely been reported.

In addition, cancer cells that obtain characteristics of stem-like properties express developmentally regulated genes associated with normal stem cells [5]. Among these crucial mechanisms, the Wnt and Notch signaling pathways have been proven to be critical for various cell events [19]. Moreover, the Wnt/β-catenin signaling pathway has been implicated in the maintenance of self-renewal in various types of stem cells through activating target genes, such as CD44, Cyclin D1, c-myc, and p21 [19]. In comparison, Notch signaling was proven to promote the differentiation of stem cells and the activation of Hes family proteins, Myc, p21, and Cyclin D3. Although accumulating data have underlined that the cooperative or adversarial relationship between the Wnt/Notch signaling pathway correlates with tumorigenesis, dormancy, and progression, their respective roles in cancer stem–like cell development remain uncertain [20]. Furthermore, the crosstalk between these pathways and its effect on therapy, particularly radiotherapy, warrants discussion. Radiosensitivity promotes cell–cell contact, which facilitates radical scavenging, DNA repair, and cell-cycle arrest [21]. In conclusion, cancer cells transform into stem-like cells via the acquisition of quiescence, and their responses to radiation might explain the persistence of radioresistance.

The purpose of this study was to investigate the effect of IL-23 on the stem-like properties of ESCC cells. To this end, we analyzed the specific characteristics of IL-23R^+^ ESCC cells to detect their capability of radioresistance after IL-23 treatment. In addition, we utilized small-molecule inhibitors or siRNA to explore the role of Wnt/Notch signaling in the development of dormancy, particularly in IL-23-mediated cell-cycle modulation.

## Materials and methods

### Cell culture

The human ESCC cell lines TE-1 was purchased from the Cell Bank of the Chinese Academy of Sciences (Shanghai, China), and TE-10, KYSE-150, and ECA-109 cells were a gift from Dr. Ye Hua of the Clinical Medicine College at Jiangsu University. The human esophageal epithelial cell line, Het-1A, was purchased from Jennio Biological Technology (Guangzhou, China). Cells were respectively maintained in RPMI 1640 or DMEM (Gibco, Carlsbad, CA, USA) containing 10% fetal bovine serum (HyClone, Logan, UT, USA) and cultured at 37 °C in 5% CO_2_. IL-23, anti-IL-23, and anti-IL-23R were purchased from R&D Systems (Minneapolis, MN, USA), and WHI-P154, DAPT, and ICG-001 were purchased from Selleck Chemicals Inc. (Houston, TX, USA). For RNA interference, cells were transfected with siRNA oligos (Gene Pharma, Shanghai, China) against human β-catenin and Notch1 using Lipofectamine 2000 (Invitrogen, San Diego, CA, USA). The following siRNA sequences (GenePharma, Shanghai, China) were used: siRNA-β-catenin: sense, 5′-GUCCUGUAUGAGUGGGAACTT-3′; antisense, 5′-GUUCCCACUCAUACAGGACTT-3′. siRNA-Notch1: sense, 5′-GGGCUAACAAAGAUAUGCATT-3′; antisense, 5′-UGCAUAUCUUUGUUAGCCCTT-3′. Negative controls using non-transfected cells and empty vector-transfected cells were performed in parallel. The colony formation assay and soft agar assay were described previously [22].

### Patient samples

ESCC and adjacent non-cancerous tissues were collected from patients at the Affiliated Hospital of Jiangsu University (Zhenjiang, China). The tumors had not been treated with chemotherapy or radiotherapy, as confirmed by the Department of Pathology of the Affiliated Hospital of Jiangsu University. Tumor staging was determined according to the American Joint Committee on Cancer criteria. Immunohistochemical (IHC) and immunofluorescence staining, scoring and statistics were performed as previously described [14], and primary antibodies for Oct-4A, CD133, IL-23, IL-23R, CD68, and HLA-DR were purchased from R&D Systems (Minneapolis, MN, USA).

### Flow cytometry and cell sorting

The number of M1 macrophages was measured in 16 independent ESCC samples and their adjacent normal samples. Surface antigens were labeled by incubation with anti-human HLA-DR/CD68 antibodies (BD Biosciences, San Jose, CA, USA). To identify CD133^+^/IL-23R^+^ cell subpopulations, ESCC cells (ESCCs) were incubated with APC-conjugated anti-CD133 (Miltenyi Biotec, Auburn, CA, USA) and PE-conjugated anti-IL-23R antibodies (R&D Systems, Minneapolis, MN, USA) or isotype control antibodies. For the cycle analysis, cells were stained with PI (Sigma-Aldrich, St. Louis, MO, USA). To identify early apoptotic cells after ablative radiation (AR, 10 Gy once) or fractionated radiation (FR, five doses of 2 Gy once every 48 h for up to 10 Gy), cells were stained with Annexin V-FITC and PI (KeyGen Biotech, Nanjing, China). The levels of ROS were determined by staining cells with DCFH-DA (Sigma-Aldrich, St. Louis, MO, USA). CFSE (Sigma-Aldrich, St. Louis, MO, USA) was diluted to a desired working concentration in normal medium to culture and label cells. The degree of DNA damage was determined based on quantity of γH2AX (Millipore, Milford, MA, USA). The data were additionally analyzed using the FlowJo software (Tree Star Inc., Ashland, OR, USA). All experiments were independently repeated at least three times.

### Molecular biology experiments

For qRT-PCR, total RNA was extracted from cells using TRIzol reagent (Invitrogen, CA, USA), according to the manufacturer’s instruction. The following primers were used to detect specific mRNAs (Invitrogen, Shanghai, China): c-myc (forward, 5′-ACCACCAGCAGCGACTCT-3′; reverse, 5′-GCTGTGAGGAGGTTTGCTGT-3′); Oct-4A (forward, 5′-GAGAATTTGTTCCTGCAGTGC-3′; reverse, 5′-GTTCCCAATTCCTTCCTTAGTG-3′); Cyclin D1 (forward, 5′-GATCAAGTGTGACCCGGACT-3′; reverse, 5′-TCCTCCTCTTCCTCCTCCTC-3′); Hes 1 (forward, 5′-TGAAGGATTCCAAAAATAAAATTCTCTGGG-3′; reverse, 5′-CGCCTCTTCTCCATGATAGGCTTTGATGAC-3′); β-catenin (forward, 5′-GCCCAGGACCTCATGGAT-3′; reverse, 5′-CCAAAATCCATTTGTATTGTTACTCC-3′); Notch1 (forward, 5′-CACTGTGGGCGGGTCC-3′; reverse, 5′-GTTGTATTGGTTCGGCACCAT-3′). A Western blot analysis was performed as previously described [14].

### Clonogenic survival assays

Clonogenic survival assays data were employed to identify radioresistance. To this end, exponentially growing cells were cultured with IL-23 (50 ng/mL) or isometric sterile PBS for 24 h and then treated with ablative radiation for different doses (0, 2, 4, 6, 8, and 10 Gy). Cells were grown for up to 9 days, then fixed and stained with 2% crystal violet; colonies of at least 50 cells were scored. Cell survival curves based on the mean survival fractions of the cell line were generated using the linear-quadratic equation according to the following formula: SF = exp ^−(*αD* + *βD*2)^, where SF is the surviving fraction and *D* is the dose.

### Methods for evaluation in vivo

ESCC xenografts were implanted subcutaneously by injecting CD133^−^IL-23R^+^ TE-1 cells (1 × 10^6^) into the dorsal anterior flank of nude mice (BALB/c inbred, female, 3–4 weeks old, *n* = 7 per group) to examine the activity of IL-23. Radiation group tumors were treated with 4 Gy of ionizing radiation every 72 h up to a total of 24 Gy, and human recombinant IL-23 (50 ng) was intratumorally injected 1 h prior to each radiation treatment. For the combined treatment, DAPT (0.2 mg) and ICG-001 (0.5 mg) were injected 6 h apart on the day before radiation through intravenous injection of the caudal vein. The growth curves were calculated along with indicated treatments. The tumor volume was measured with a Vernier caliper and calculated as following: tumor size = (π × length × width × height)/6. Radiation was administered using the small animal radiation resource platform, and tumor volume curves were plotted every week. Xenografts were harvested for molecular analysis, and the standard deviation values were determined for each sample’s normalized values.

### Statistical analysis

The differences between groups in the clone assay, flow cytometry, protein expression, RT-PCR, and animal experiments results were analyzed using one-way or two-way ANOVA. Survival curve were analyzed by the Kaplan–Meier method. The difference was significant for *p* values of < 0.05. All data were analyzed using the SPSS version 16.0 software (Chicago, IL, USA).

## Results

### IL-23/IL-23R is correlated with the acquisition of stem-like potential in ESCC

**Fig. 1:**
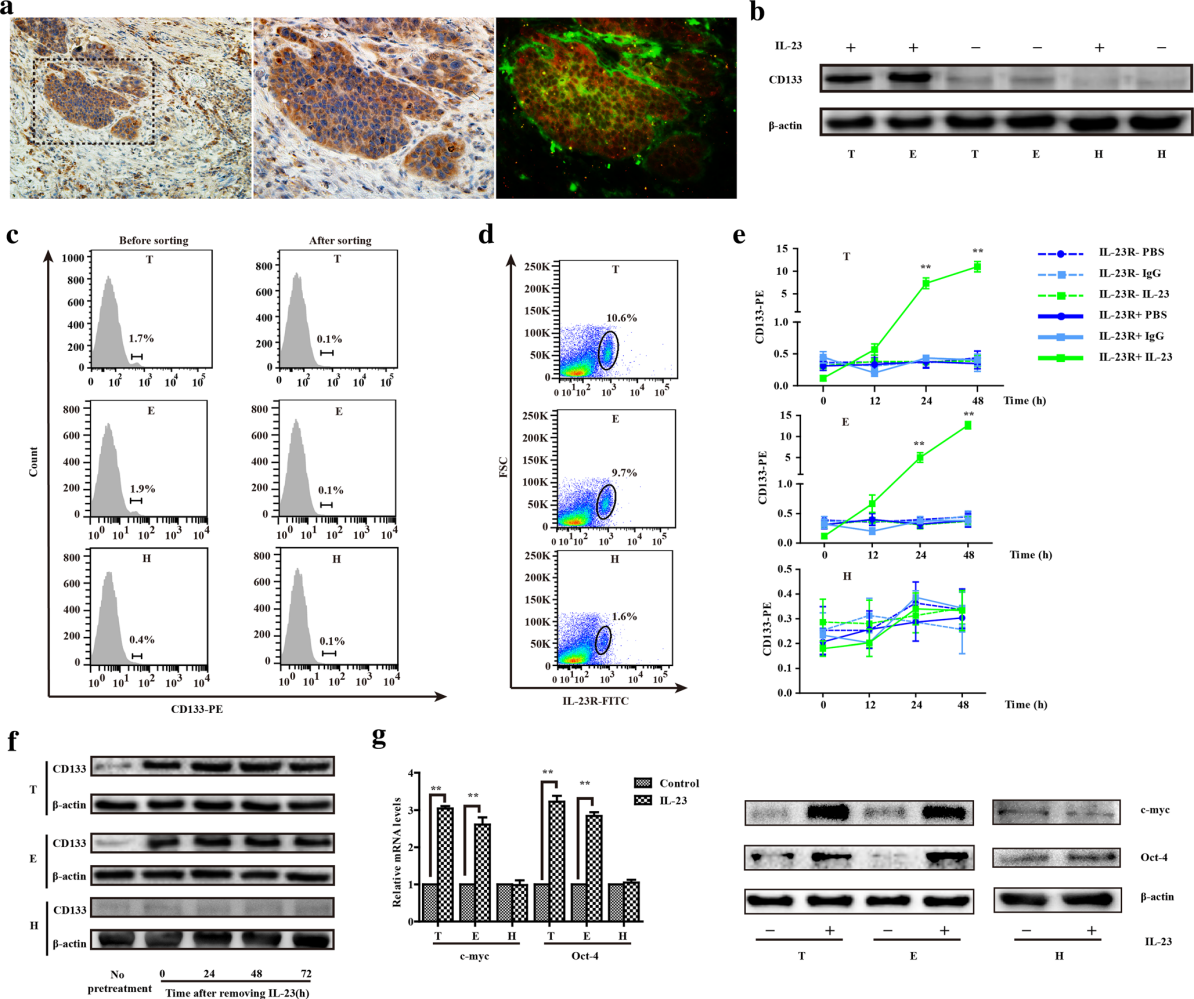
The correlation between IL-23/IL-23R and the trans-differentiation of ESCCs. **a** Typical immunofluorescence images of the distribution of Oct-4A^+^ cells (red) and IL-23R^+^ ESCCs (green) gathering with the intensity of IL-23 (IHC). Left panel, × 200 magnification. Middle and right panels are magnifications of the area marked by dashed lines. **b** Western blotting: the expression of CD133 in ESCC cells (TE-1, ECA 109, KYSE 150, and TE-10) and Het-1A cells with or without IL-23 treatment (50 ng/mL, 24 h). The results were normalized to β-actin as a control and densitometric analysis of bands was performed with Alpha View. T, TE-1 cells; E, ECA 109 cells; H, Het-1A cells. **c** The percentage of CD133^+^ cells in ESCCs and Het-1A cells before and after sorting. Flow cytometry and fluorescent cell sorting were performed using anti-CD133 fluorescent-labeled antibody. The representative experiment results were compared with that untreated groups. **d** The number of protogenetic IL-23R^+^ cells. Flow cytometry was performed using anti-IL-23R (FITC) in ESCCs and Het-1A cells. The experiment was repeated twice and representative data shown. **e** The variants of CD133^+^ cells between IL-23R^−^/IL-23R^+^ CD133^−^ESCCs and Het-1A cells cultured with IL-23 (50 ng/mL, 24 h). **f** CD133^−^IL-23R^+^ ESCCs and Het-1A cells were pretreated with IL-23 (50 ng/mL) for 24 h, the expression levels of CD133 were detected by Western blotting at 0, 24, 48, and 72 h after removing IL-23. **g** The relative mRNA expression levels of stemness genes (c-myc and Oct-4A) were measured by RT-PCR in CD133^−^IL-23R^+^ ESCCs and Het-1A cells cultured with IL-23 (50 ng/mL) for 24 h. The GAPDH was used as the loading control. TE-1, ECA 109 cells, and Het-1A cells were treated with IL-23 for 48 h or not, and the protein expression of Oct-4A and c-myc was determined by Western blot analysis. β-Actin was used as a loading control. The data are presented as the mean ± SD from at least three independent experiments. **p < 0.01

We first examined IL-23 expression in 56 tumor tissue sections from patients with ESCC by immunohistochemistry. The results showed that high-intensity IL-23 clustered in the vessels, surrounding small lymph nodes, the edges of tumors, and areas infiltrated by cancer cells in the tumor tissues (Fig. 1a and Supplementary Fig. 1). IL-23 expression was sporadic and overall lower in control biopsy tissues from donors who were diagnosed with reflux esophagitis (Supplementary Fig. 1E). Remarkably, IL-23 was also high in para-carcinoma tissues, but the significant expression difference between tumors and para-carcinoma tissues further validated our previously published work (Supplementary Table 1) [14]. M1 macrophages, the primary source of IL-23, are also known as tumor-associated macrophages (TAMs) [23]. Although the number of HLA-DR^+^CD68^+^ cells, defined as M1 macrophages population, did not markedly change during tumor development in this study, and more activated M1 macrophages were detected in the pathological tissue than in para-carcinoma tissue (Supplementary Fig. 2A). In addition, the expression of Oct-4A, a marker of self-renewal, undifferentiated stem cells or poor prognosis for patient with cancers, co-localized with the IL-23R^+^ ESCCs (Fig. 1a) [19]. These results suggested that IL-23 might indicate stem-like properties of ESCCs. To confirm this, IL-23 was used to treat the ESCCs, which significantly increased the expression of CD133, another marker of stem-like properties. However, this treatment did not affect CD133 expression by the Het-1A cells. As the initial element, the baseline expression level of IL-23R was not significantly different between cancer cell lines and remained relatively constant, albeit weak in Het-1A cells before and after treatment with IL-23 (Fig. 1b and Supplementary Fig. 2B, C).

To eliminate the potential effects of innate CD133^+^ cells, we isolated CD133^−^ cells from TE-1 and ECA 109 cells or Het-1A cells using FACS. The mean proportions of cells that expressed CD133 were approximately 1.7% for TE-1 cells and 1.9% for ECA 109 cells, which is almost invisible in Het-1A cells (Fig. 1c). Moreover, a flow cytometry analysis showed that about 8 to 10% of CD133^−^ ESCCs and less than 3% of Het-1A cells were IL-23R^+^ cells (Fig. 1d). Therefore, after sorting the CD133^−^IL-23R^−^/ CD133^−^IL-23R^+^ cells, we found that more CD133^+^ cells were detected in the CD133^−^IL-23R^+^ ESCCs after culturing with IL-23 (Fig. 1e). In addition, the expression of CD133 was upregulated in CD133^−^IL-23R^+^ cells cultured with IL-23. Meanwhile, the CD133^−^IL-23R^+^ cells were pretreated with IL-23 for 24 h, and the acquired upregulation of CD133 expression was maintained for more than 72 h after the removal of IL-23 in ESCCs (Fig. 1f). Furthermore, we demonstrated that IL-23 induced the expression of the aforementioned Oct-4A and c-myc, which are transcription factors involved in cellular transformation and cell cycle progression (Fig. 1g). We have previously suggested that aberrant IL-23 expression enhances the activity of EMT in ESCCs [14]. Therefore, IL-23 mediates the trans-differentiation not only by affecting the accumulation of stem-like makers but also by directionally inducing the expression of stemness-related genes in IL-23R^+^ ESCCs.

### CD133^−^IL-23R^+^ ESCCs acquired partial stem-like properties especially dormancy via IL-23

**Fig. 2:**
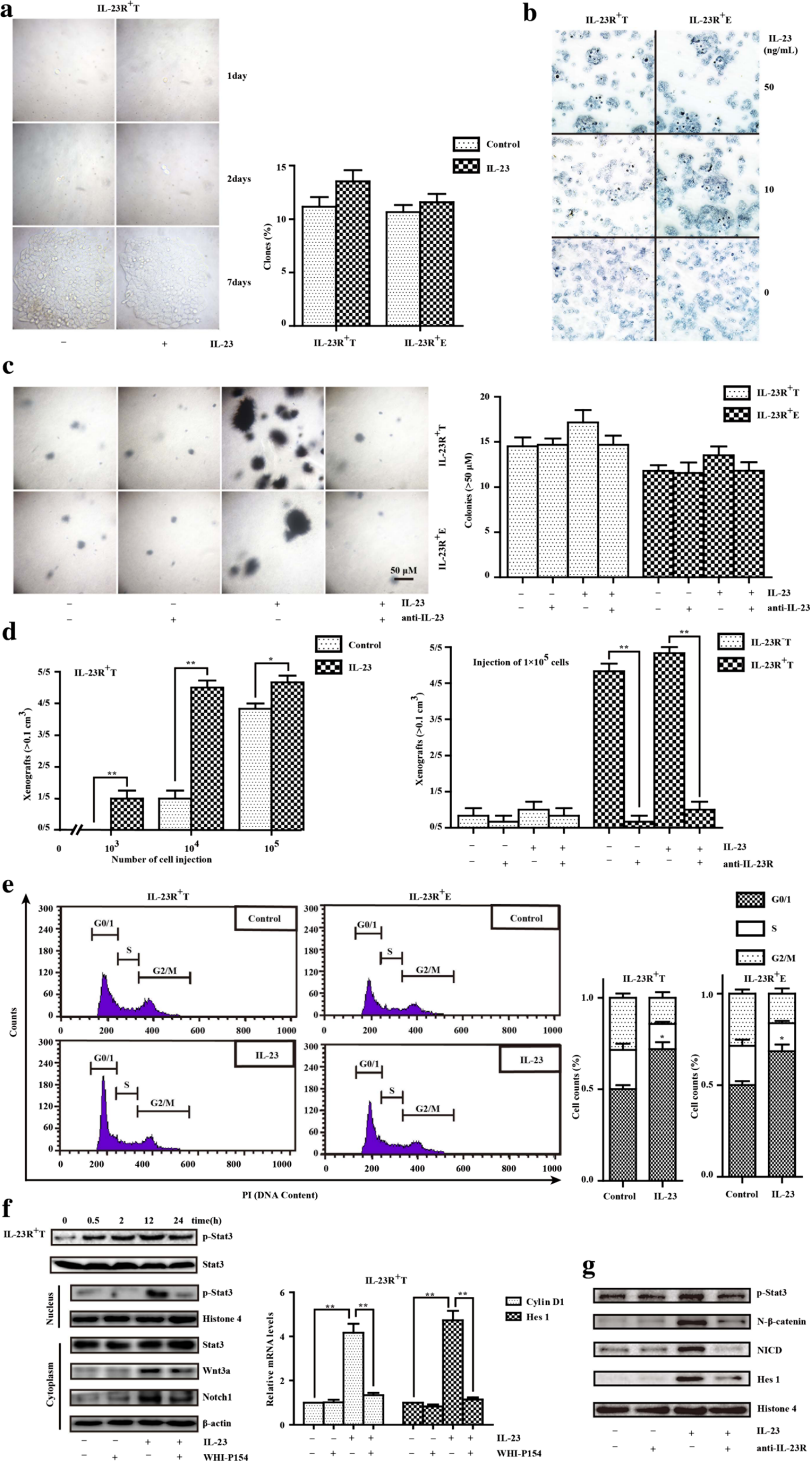
The stem-like potencies of CD133^−^IL-23R^+^ ESCCs stimulated by IL-23. **a** The plate clonogenic assay was performed in ESCCs. The CD133^−^IL-23R^+^ TE-1 and ECA 109 cells were cultured with IL-23 (50 ng/mL) or not for 7 days. IL-23 had no significant impact on the clonogenicity ability of TE-1 cells (left panel). The statistical results (right panel) are the mean ± SEM (*n* = 3). **b** After the IL-23 treatment (10 or 50 ng/mL, 24 h) or not, CD133^−^IL-23R^+^ TE-1 and ECA 109 cells expressing alkaline phosphatase (AKP) were counted. **c** The tumorigenic potentials with IL-23 treatment in CD133^−^IL-23R^+^ TE-1 and ECA 109 cells were measured by the soft agar assay. Cells were cultured for 10 days in serum-free DMEM in the presence or absence of IL-23 (50 ng/mL) and/or IL-23 antibody (1 μg/mL). The results are the mean ± SEM (*n* = 3) (right panel). **d** The pretreatment of anti-IL-23R (0.5 μg) reversed the significant difference between the injection of IL-23 (50 ng) or vehicle groups that comprised IL-23R^+^ TE-1 cells (left panel). CD133^−^IL-23R^+^ TE-1 cells were transplanted into nude mice in serial limiting dilutions (100,000, 10,000, or 1000 cells per injection) to form xenografts (> 0.1 cm^3^). **e** Phase of cell-cycle analysis by flow cytometry showed that IL-23R^+^ ESCC arrest at G0/1 correlated with IL-23 (50 ng/mL, 24 h) in CD133^−^IL-23R^+^ TE-1 and ECA 109 cells. **f** The protein expressions of p-Stat3/Stat3, Wnt 3a, Notch 1, and the activation of the Wnt and Notch pathways (Cyclin D1 and Hes 1) were assessed by Western blotting and RT-PCR. The Western blotting results were normalized to β-actin or Histone 4 as the control. Cells were pretreated with WHI-P154 (2 μM) 2 h before adding IL-23 (50 ng/mL). IL-23 treatment (50 ng/mL, 24 h) activated Wnt/Notch signaling and the persistent phosphorylation level of Stat3 was maintained up to 24 h in TE-1 cells (left panel). Relative mRNA level of Cyclin D1 and Hes 1 were detected by RT-PCR in TE-1 cells (right panel). Compiled data were produced from three independent experiments. **p < 0.01. **g** Western blotting assessed the proteins expression of Stat3, Wnt, and Notch pathway. Cells were pretreated with anti-IL-23R (1 μg/mL) 2 h before adding IL-23 (50 ng/mL). The Western blotting results were normalized to β-actin or Histone 4 as the control

First, the plate clonogenic assay showed that the number of spherical colonies formed by CD133^−^IL-23R^+^ TE-1 and ECA 109 cells incubated with IL-23 for 7 days (13.7 ± 2.4% and 11.9 ± 1.0%) did not differ from those formed by the respective control cells (10.3 ± 0.3% and 11.4 ± 0.1%) (Fig. 2a). Furthermore, IL-23 treatment did not increase the number of alkaline phosphatase (AKP)–positive cells, and this finding depends on the AKP negativity of re-differentiated stem cells (Fig. 2b) [24]. Compared with the untreated cells, IL-23 increased the number of colonies formed by CD133^−^IL-23R^+^ ESCCs, with a mean number of 17 and 14 colonies per visual field, respectively, but this difference was not significant. Moreover, these weak effects were blocked by anti-IL-23 treatment (Fig. 2c). To observe the effect of IL-23 on tumor-initiating potential, we transplanted CD133^−^IL-23R^+^ TE-1 cells into nude mice in serial limiting dilutions to form xenografts. Injections of more than 1 × 10^4^ cells formed 0.1 cm^3^ xenografts within 7–12 days when exposed to IL-23, but this effect was restrained by anti-IL-23R treatment. Comparably, 1–5 × 10^6^ IL-23R^−^ cells are required with or without IL-23 treatment to form tumors of approximately the same volume (Fig. 2d).

As mentioned, most cancer cells with stem-like properties are characterized by cell-cycle arrest at G0/1. IL-23 was reported to promote proliferation in various types of cancers, but not in ESCCs, according to our previous study [14]. Specifically, treatment with IL-23 rapidly increased the percentage of cells arrested at G0/1 in TE-1 (from 50.10 ± 5.22% to 71.90 ± 9.44%) and ECA 109 cells (from 49.96 ± 5.34% to 68.57 ± 9.01%), compared to controls (Fig. 2e). As transcription factors that link the immune signaling and tumor activity, STAT proteins regulate the key aspects of cancer cell growth, survival, and differentiation [25]. Our data identified excessive p-Stat3 expression in the cells when exposed to IL-23. Moreover, the accumulation of p-Stat3 has been revealed that promotes dysfunction in NF-κB, Wnt, Notch, or other cell signaling pathways in a variety of cells [26]. Accordingly, we found that treating IL-23R^+^ ESCCs with extracellular IL-23 stimulated the expression of Wnt3a (> 3.0-fold change, *p* < 0.01), Notch1 (> 1.9-fold change, *p* < 0.05), Cyclin D1(the mRNA level, *p* < 0.01), and Hes-1(*p* < 0.01), and this effect was attenuated by WHI-P154 (inhibitor of Stat3 phosphorylation, *p* < 0.01 at both ratios), while the pretreatment of anti-IL-23R blocked the phosphorylation cascades and activated Wnt/Notch that trigged by IL-23 (Fig. 2f, g). Taken together our data indicate that the activation of Stat3 is required for IL-23 binding to IL-23R and that Wnt and Notch may work in a combinatorial manner in the regulation of tumorigenesis and dormancy.

### Radioresistance depends on IL-23-mediated Wnt/Notch pathway activation

**Fig. 3:**
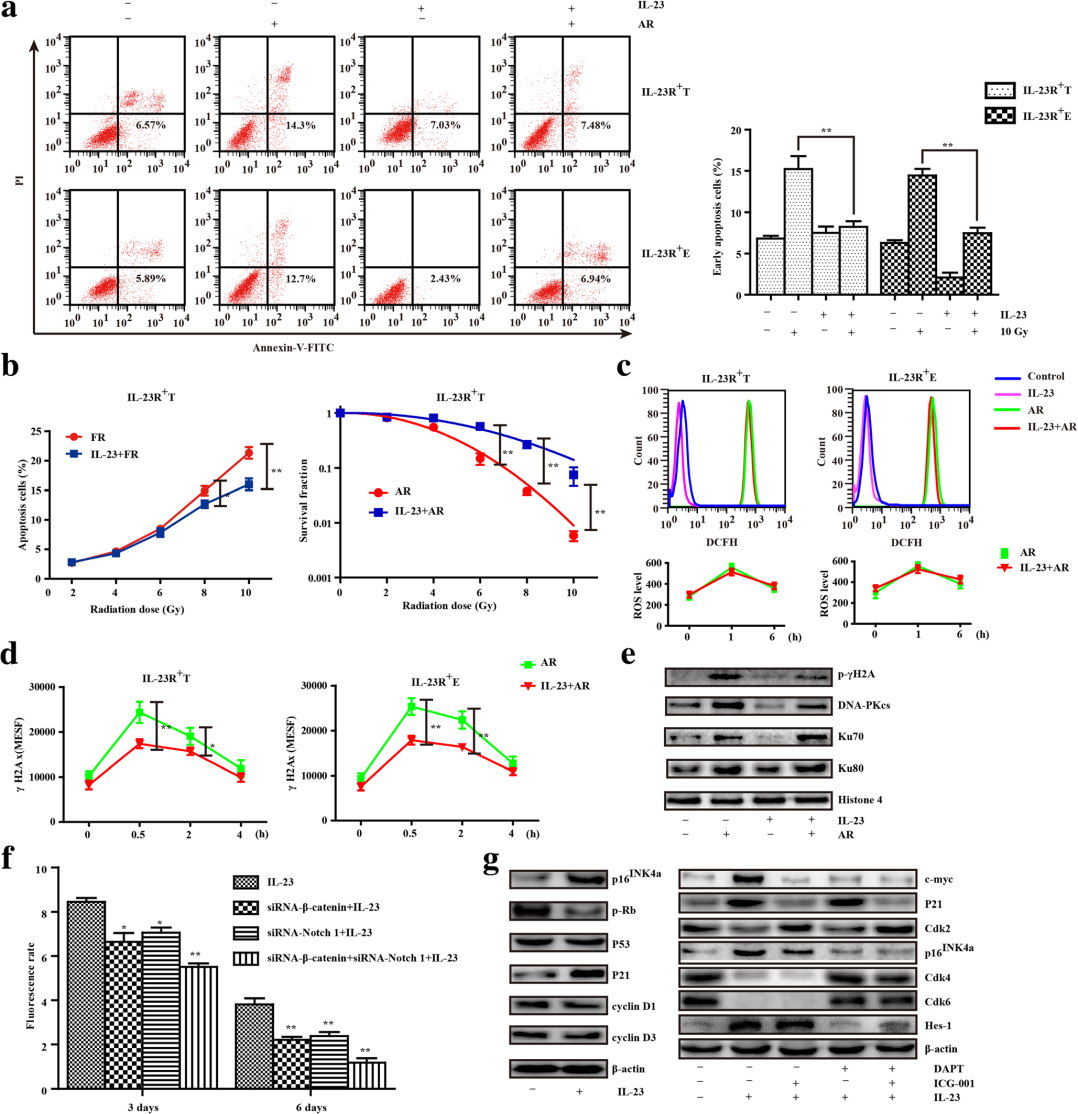
The Wnt/Notch signaling-mediated cell-cycle arrest due to IL-23 resulted in radioresistance. **a** The representative percentage of early apoptotic CD133^−^IL-23R^+^ TE-1 and ECA 109 cells evaluated by Annexin-V and PI uptake after exposure to 10 Gy of ablative radiation (AR) with or without IL-23 treatment (50 ng/mL, 24 h) (left panel). The results are the mean ± SEM (*n* = 3, **p < 0.01) (right panel). **b** Early apoptotic CD133^−^IL-23R^+^ TE-1 cells after each exposure to FR (2 Gy per fraction up to 10 Gy) with or without IL-23 treatment (50 ng/mL, 48 h) (left panel); SF of colonies were counted after cells exposed to AR (0, 2, 4, 6, 8, 10 Gy) with or without IL-23 treatment (50 ng/mL, 24 h) (middle panel). **c** According to the DCFH-DA fluorescence level by flow cytometry, the evaluation of cellular ROS levels after the exposure of CD133^−^IL-23R^+^ ESCCs to AR (0, 1, and 6 h) with or without IL-23 (50 ng/mL, 24 h). **d** Formation of γH2AX was visualized in CD133^−^IL-23R^+^ ESCCs after 10 Gy of AR (0, 0.5, 2, and 4 h) or pretreatment with IL-23 (50 ng/mL, 24 h). **e** The expression of p-γH2A, DNA-PKcs, Ku70, and Ku80 were detected in CD133^−^IL-23R^+^ TE-1 cells cultured with IL-23 (50 ng/mL, 24 h) or not which were exposed to 10 Gy of AR by Western blotting. **f** CD133^−^IL-23R^+^ TE-1 cells were transduced with non-silencing control siRNA (data not shown), siRNA-β-catenin, and/or siRNA-Notch1before the treatment of IL-23 (50 ng/mL). Cell proliferation was assessed by flow cytometry for CFSE labeling on the third and sixth days. **g** The protein expression changes of the cell cycle–regulating factors and Wnt/Notch signaling-related proteins from the IL-23 treatment (50 ng/mL, 24 h) and/or inhibitors (DAPT at 2 μM; ICG-001 at 5 μM) were detected by Western blotting. Compiled data were produced from three independent experiments. **p < 0.01

To assess the ability of IL-23 to mediate radioresistance accordingly, we traced morphological changes and analyzed cell death after irradiation. Marked cell death was observed within AR, and more cells exhibited a vacuolated cytoplasm, abnormal nuclei, bareness, and debris with moribund flatness (data not shown). In contrast, IL-23 pretreatment significantly decreased the percentage of cell apoptosis in CD133^−^IL-23R^+^ TE-1 cells (8.25 ± 1.67% vs. 15.22 ± 3.85%) and ECA 109 cells (7.47 ± 1.64% vs. 14.45 ± 1.92%), compared with AR alone (Fig. 3a). To mimic clinical therapy, CD133^−^IL-23R^+^ TE-1 cells were exposed schedule of FR. One day after each exposure, data from an early apoptotic assay indicated that FR increased the proportion of early apoptotic cells. However, IL-23 significantly inhibited this apoptosis from 21.35 ± 2.39% to 16.02 ± 2.46%, particularly after the fourth and fifth dose of FR, whereas the SF of colonies stained with crystal violet did not significantly differ from that of cells until treated with 6 Gy of AR (*p* = 0.002) (Fig. 3b).

Abnormal DNA repair and radicals scavenging capacity as well as cell-cycle arrest are talismans of cancer stem-like cells exposed to radiation [21, 24]. Although reactive oxygen species (ROS) only differ between groups exposed to irradiation or not (Fig. 3c), IL-23 markedly suppressed the level of γH2AX in CD133^−^IL-23R^+^ ESCCs compared to cells treated with AR alone (Fig. 3). As shown in Fig. 3e, radiation induced the phosphorylation of γH2AX and increased the expression levels of DNA-PKcs, Ku70, and Ku80 within 1 h. However, pretreatment with IL-23 did not influence the accumulation of these factors except p-γH2AX. In addition, knocking down β-catenin or Notch 1 gene expression significantly inhibited the IL-23-induced arrest of CD133^−^IL-23R^+^ TE-1 cells, as detected by flow cytometry of CFSE labeling (cells co-transfected for 3 days, 55.17 ± 3.71%; NC cells, 84.50 ± 4.09%; siRNA-β-catenin cells, 66.50 ± 9.67%; and siRNA-Notch1 cells, 70.67 ± 5.72%; cells co-transfected for 6 days, 11.83 ± 5.08%; Fig. 3f). To further verify that IL-23 regulates cell-cycle arrest via the Wnt/Notch signaling pathway, we measured the activation and increase in proteins expression at the G0/1 and G1/S checkpoints, and added ICG-001 (binding to CREB-binding protein) and DAPT (a γ-secretase inhibitor) to CD133^−^IL-23R^+^ ESCCs. The key regulator p21 was significantly upregulated in response to IL-23 treatment, whereas the level of Cdk2 decreased, which arrested cells in the S phase that was blocked by ICG-001. After cultured with IL-23, increased p16^INK4a^ expression and downregulated Cdk4/6 expression were observed, which arrested most cells at the G0/1 phase. However, the above changes in p16^INK4a^ and Cdk4/6 were abrogated when cells were pretreated with ICG-001 or DAPT, and IL-23 downregulated the phosphorylation of Rb but no longer produced significant changes in p53, Cyclin D1, and Cyclin D3 expression (Fig. 3g). Thus, we found that IL-23 could induce IL-23R^+^ ESCCs into dormancy and ultimately radioresistance.

### IL-23 mediates radiation resistance in human ESCC xenografts

**Fig. 4:**
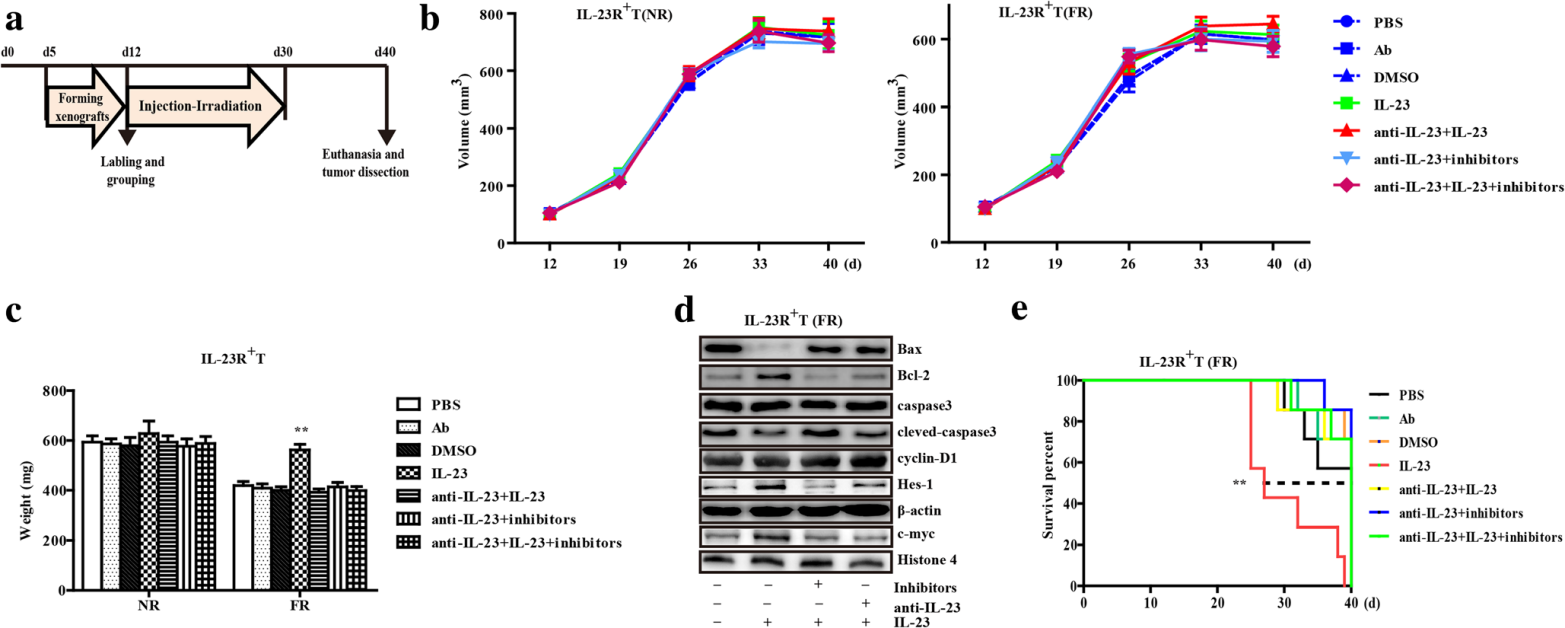
The in vivo study of IL-23 and radioresistance in a xenograft mouse model of ESCC. **a** Graphical representation of the experimental protocol and treatment schedule for stimulation and radiation in mice. **b** Line chart represents the average periodical tumor volume every week. The growth curves of CD133^−^IL-23R^+^TE-1 cell xenograft tumors receiving different treatments, including PBS, isotype Ab, DMSO vehicle, irradiation, anti-IL-23 (1 μg in 100 μL PBS), human recombinant IL-23 (50 ng in 100 μL PBS), and the composite inhibitors (DAPT, 20 μmol and ICG-001, 50 μmol dissolved with DMSO in 100 μL PBS per day). The tumor volume was calculated as described in the “Materials and methods” section. NR, no-irradiation treatment; FR, fractionated radiation. **c** Column chart of xenograft tumor weights after different treatments (PBS, anti-IL-23, IL-23, irradiation, and the inhibitors). The tumors were weighed after 40 days or death. **d** Immunoblot analysis of activated Wnt/Notch signaling and apoptosis-related proteins in homogenized xenograft lysates after radiotherapy. After 40 days, the survived mice were euthanized, and tumors were harvested from all groups to assess the effect of inhibitors. Representative data are shown. **e** Fractions of surviving animal in different groups (*n* = 7 per group). **p < 0.01

Because our cellular assays showed that IL-23 was involved in the maintenance of the tumorigenicity and Wnt/Notch-derived cycle-regulation progression, we explored the therapeutic value of signaling inhibitors. In consideration of the weak immunogenicity of these mice, extra recombinant human IL-23 and/or mouse anti-IL-23 (the IL-23-p19 subunit of humans and mice share nearly 70% amino acid sequence identity) was injected into the tumors to verify that mouse IL-23 resulted in negligible interference in this study (data not shown). The inhibitors were administered six times every 3 days. On the last day of each cycle, the mice received 4 Gy of total-body irradiation with integration to 24 Gy. All mice were then sacrificed to remove the tumors for further analysis and the detailed process is summarized in Fig. 4a. Moreover, the tumors treated with IL-23 showed no evident volume changes, but once exposed to FR, the significant decreased weight occurred with or without inhibitors and anti-IL-23 (Fig. 4b, c). We also validated that the changes of the Bax and cleaved caspase-3 were downregulated by IL-23, corresponding with the significant change of Wnt/Notch signaling, while Bcl-2 was regenerated after treatment with inhibitors in the subcutaneous xenografts (Fig. 4d), which indicated the specificity of the resistance by IL-23 that depended on the activation of Wnt/Notch. Finally, we investigated the effect of inhibitors on the significant tumorigenic difference and overall survival time in IL-23 stimulated tumor-bearing mice (Fig. 4e and Supplementary Fig. 3). In short, the Wnt/Notch inhibitors effectively attenuated radioresistance caused by IL-23.

## Discussion

Comparisons and analyses of the number of stem-like or CD133^+^ cells in tumor and cancer cell lines have confirmed the existence of the innate cancer stem-like cells [5, 6, 8, 13]. Dormancy that prevents normal stem cell exhaustion is characteristic of cancer stem-like cells and allows proliferation to resume, and also regulates proliferation and differentiation in the tumor [11, 18, 27]. By entering a quiescent state, cancer cells can survive therapy. Moreover, tumor dormancy is a state of clinical remission, and a quantitative analysis of ovarian tumors showed that only approximately 30% of all tumor cells progressed through the cell cycle; similar observations have been made in melanoma, prostate, and breast cancer [28–31]. Recent studies show that the existing immunotherapies primarily arrest the growth instead of eliminating the cancer, and immuno-environmental disturbances are not only involved in the malignant pathology of tumors but also directly and continuously obstruct the therapeutic efficacy [18]. Among this complex network, TAMs are closely related to tumor masses from either circulating monocytes or tissue-resident macrophages, which clearly interact with a wide range of growth factors and cytokines, such as IFN-γ, IL-6, and IL-23, even their function is controversial. Our data revealed that M1 macrophages infiltrated ESCC tissues, and IL-23 exacerbated the malignancy of IL-23R^+^ ESCCs. As confirmed in our previous study, excessive IL-23 levels result in EMT, and the biological link between EMT phenotypes and cancer stem-like cells or cancer stem cells (CSCs) has recently been evidenced in many types of cancer, and a recent study of prostate cancer showed that CSCs undergo EMT to become resistant to radiation therapy [14, 32]. In the present research, the binding of IL-23 to IL-23R^+^ ESCCs strongly activated p-Stat3, which resulted in a signaling cascade that supported stem-like features, such as Wnt and Notch signaling. In contrast, Het-1A cells are more invalid and inertia to the same IL-23 treatment probably for the lower population of IL-23R. The observed self-renewal, tumorigenicity, and dormancy confirms IL-23 as an indispensable factor in the tumor microenvironment. Notably, not all IL-23R^+^ cells can be transformed into CD133^+^ cells. In other words, we concluded that the formation of stem-like properties in ESCCs is inextricably linked to the quantity of internal signal influence.

Our previous results suggested that exogenous IL-23 influenced the GSK-3β-mediated distribution of β-catenin [14]. According to other research, the levels of Wnt1 and β-catenin in ESCC patients correlate with advanced TNM staging, lymph node involvement, and poor prognosis [33]. Our study demonstrated that IL-23 not only contributed to the accumulation of β-catenin in the nucleus but also upregulated downstream genes, such as c-myc and Cyclin D1. Although Wnt signaling mainly plays a role in the maintenance of adult stem cells, recent studies have explored the integral role of Wnt signaling in cancer stem-like cells [19, 20]. However, in specific environments, Notch, which was identified to be mutually regulated by Wnt, participates in the stemness and differentiation [34]. For example, Notch signaling is likely to inhibit specific differentiation lineages and to maintain the sustainability of self-renewal tumor populations in intestinal epithelial cells, hematopoietic stem cells and esophageal adenocarcinoma cells, and these features are characteristics of stemness [35–37]. Moreover, some studies have indicated that Notch1 might be a molecular prognostic marker and an effective therapeutic target for patients with ESCC [38]. Conversely, other studies suggested that Notch1 is a tumor suppressor gene in the esophagus [39]. Interestingly, we found that IL-23 consistently stimulated Notch signaling, which was accompanied by apparent increases in p16^INK4a^ and Hes1. Specifically, the molecular interactions of the Wnt and Notch pathways initiated by IL-23 are likely to be cell-context specific and may be a unique model for resistance.

The clinical outcome of radiation and response to immunogenicity, or biomarkers predicting radiation for tumor has revealed the networks of DNA damage response and repair and immune response signaling, and the activation of response and repair triggers innate and adaptive immune response, leading to cell-cycle arrest and immune defects [40]. CSCs, cancer stem-like cells, or therapy-resistant cells in tumors are undoubtedly difficult to destroy, but all of these cells play a part in the regulation of the cell cycle. In other words, inhibiting the proliferation and reducing the exposure to free radicals is a superior approach that minimizes the damage of radiation to cancer cells. Here, we demonstrated the role of IL-23 in G0/1 phase arrest of IL-23R^+^ ESCCs; specifically, Notch induction led to cell cycle progression before the S phase, whereas Wnt resulted in cells at the G0/1 phase, and ultimately reduced apoptosis caused by radiation. Thus, we inferred that IL-23 could induce IL-23R^+^ cells into dormancy with stem-like cell markers rather than typical CSCs. The “love–hate struggle” of immune microenvironment and cancer cells is intertwined already; to be sure, the tumor also contains other stroma cells. Interestingly, irradiated fibroblasts can recover IL-23 secretion from irradiated dendritic cells through COX2-dependent PGE2 release [41]. Furthermore, previous studies showed that IL-23 increased the abundances of intercellular adhesion molecule-1 and vascular cell adhesion molecule-1 on endothelial cells, and improved the endothelial marker CD31 in mammary cancer [42, 43]. In contrast, our findings reveal no significant difference in fibroblast and endothelial cells between indicated treatments, no matter if IL-23 treatment or irradiation, in xenograft models (Supplementary Fig. 4). The above observations suggest that the activation of Wnt and Notch signaling by IL-23 resulted in the stem-like properties of ESCCs, which led to self-renewal, tumorigenesis, prominent G0/1 phase arrest, and resistance to irradiation. Thus, selectively targeting cancer stemloids might improve the therapeutic response of patients with cancer.

## Electronic supplementary material

ESM 1 (DOCX 4118 kb)

ESM 2 (DOC 57 kb)
