# Supplementary material for: Interleukin-23 receptor signaling mediates cancer dormancy and radioresistance in human esophageal squamous carcinoma cells via the Wnt/Notch pathway
Source: J Mol Med (Berl). 2018 Nov 27;97(2):177–88. doi: 10.1007/s00109-018-1724-8 (PMC6348073; doi:10.1007/s00109-018-1724-8)
Supplement: Supplementary file 1 — (DOCX 4118 kb) [file 109_2018_1724_MOESM1_ESM.docx]

**Interleukin-23 receptor signaling-mediated cancer** **dormancy triggers radioresistance in human esophageal squamous carcinoma cells via the Wnt/Notch pathway**

**Yuepeng Zhou^1*^, Yuting Su^1*^, Haitao Zhu^2^, Yu Zhu^1^, Ge Hu^1^, Xuefeng Wang^3^, Xiaoqin Li^1^, Chunhua Dai^1^, Chengcheng Xu^3^, Tingting Zheng^3^, Chaoming Mao^1, 3^, Deyu Chen^1^**

^1^ Institute of Oncology, Affiliated Hospital of Jiangsu University, Zhenjiang, 212001, China; ^2^Department of Medical Imaging, Affiliated Hospital of Jiangsu University, Zhenjiang, 212001, China; ^3^Department of Nuclear Medicine, Affiliated Hospital of Jiangsu University, Zhenjiang, 212001, China

^*^ These authors contributed equally to this work.

Corresponding author: Deyu Chen or Chaoming Mao, Institute of Oncology, the Affiliated Hospital of Jiangsu University, Jiefang Road 438, Zhenjiang, 212001, China; Phone: (86) 511-85037530; Fax: (86) 511 85037530; jq1001@ujs.

**Supplementary Figures**

**
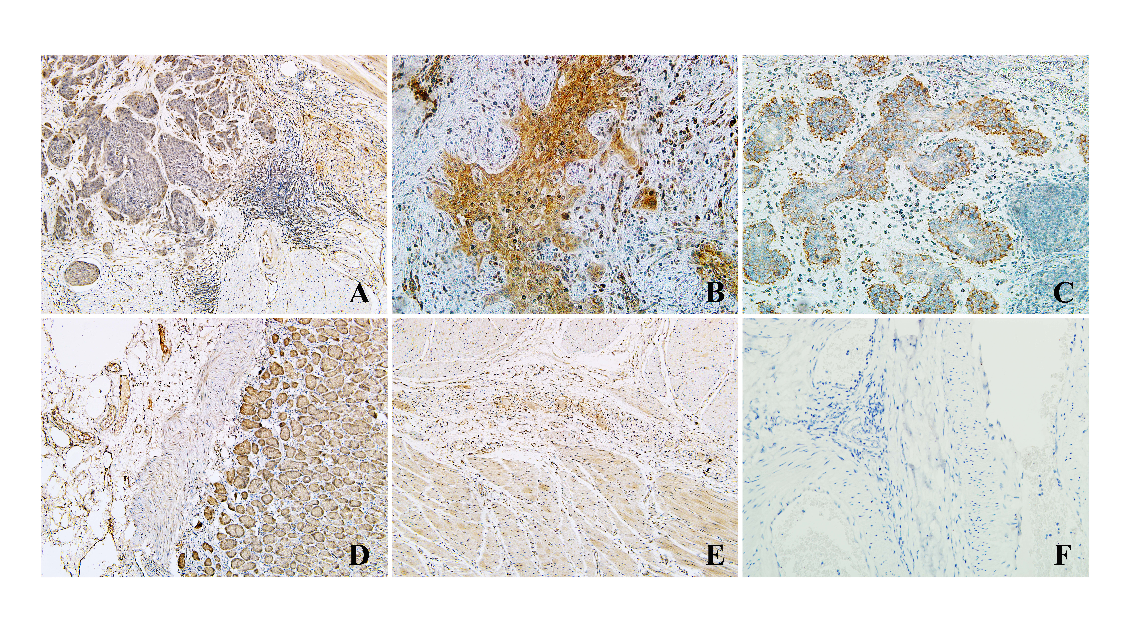
**

**Figure S1.** Representative distribution and intensity of IL-23 in esophageal squamous carcinoma or normal tissue. Both cancer nests (A, B and C) and their surrounding stroma (D) exhibited increased IL-23 immunoreactivity (brown) compared with the corresponding normal esophageal squamous tissue (5 cm away from the tumor margin) sections from 56 patients with ESCC. E. Representative figure of biopsy tissue from patient with reflux esophagitis. F. Negative control. A, D, and E, 200× magnification. B, C and F, 400× magnification.


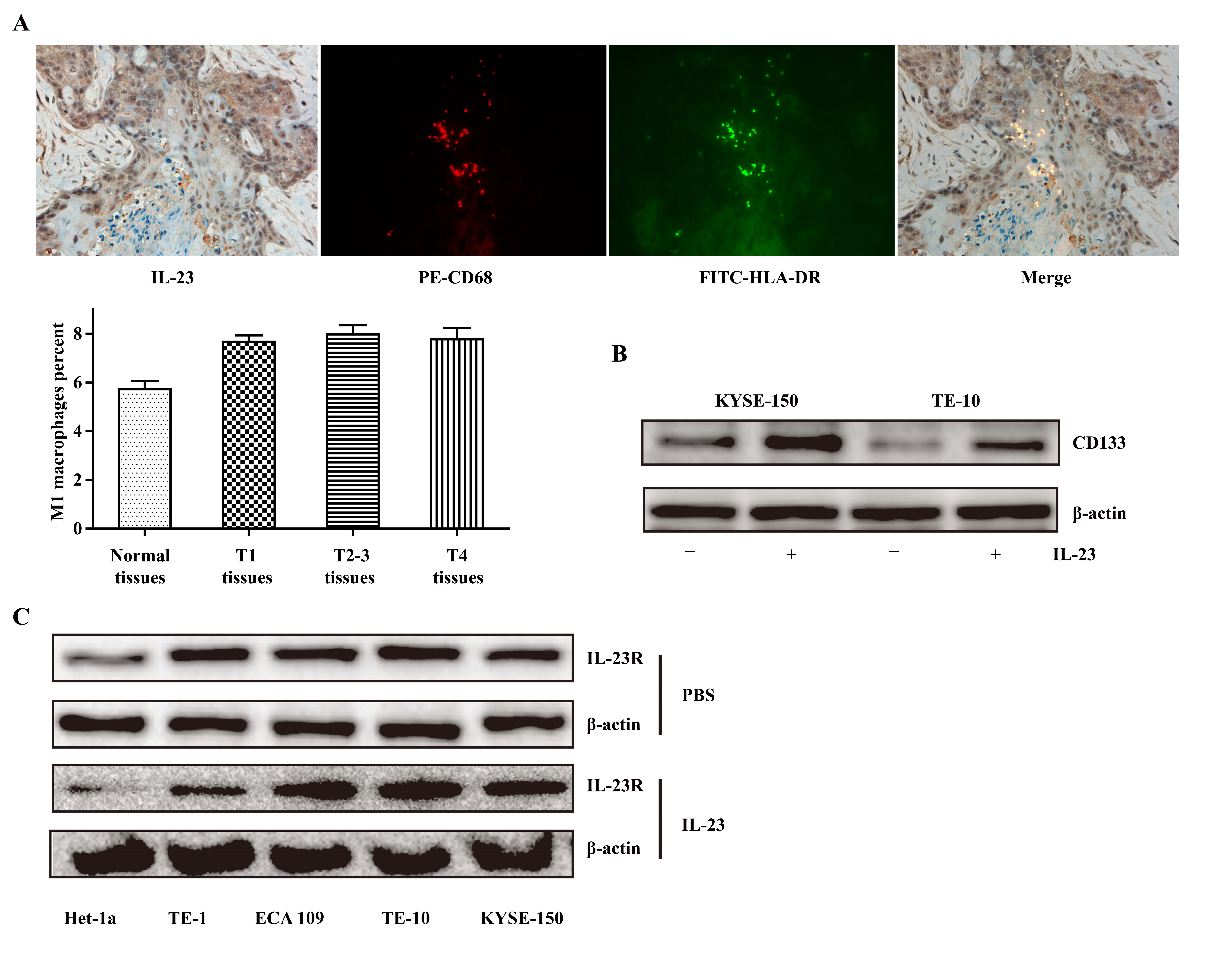


**Figure S2.** A. Representative figures of M1 macrophages (CD68^+^HLA-DR^+^ cells) and IL-23 colocalization (Brown: IL-23; Red: CD68; Green: HLA-DR) in ESCC tissues, 400× magnification. Flow cytometric validation of M1 macrophages between normal tissues and esophageal squamous carcinoma tissues at different stages. The analysis data from 4 cases of T1, 7 cases of T2/3 and 5 cases of T4 ESCC patients compared to their adjacent normal tissues respectively. B. The expression of CD133 in ESCC cells (KYSE-150 and TE-10) with IL-23 (50 ng/mL) for 24 h were assessed by immunoblotting. C. The IL-23R levels of ESCC cells (TE-1, ECA 109, TE-10, and KYSE 150) and Het-1a cells before and after IL-23 treatment (50 ng/mL, 24 h).

**
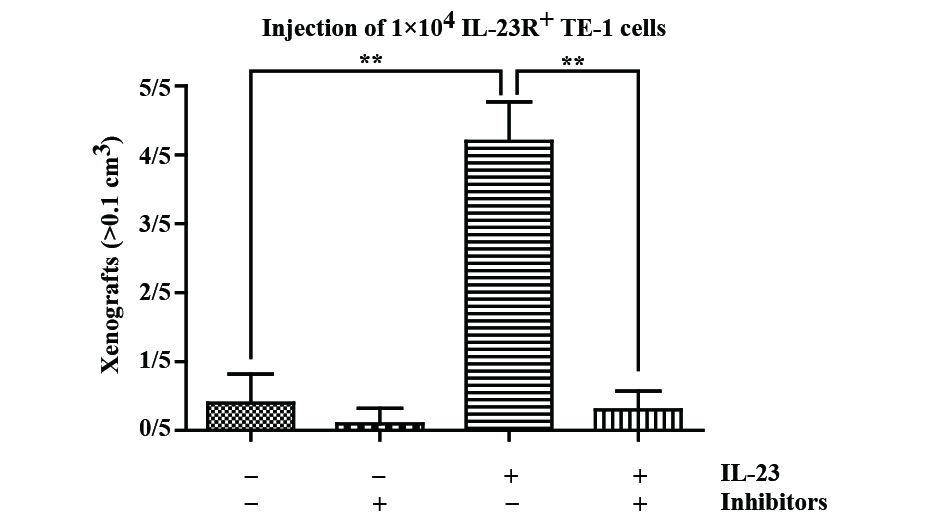
**

**Figure S3.** The pretreatment of combined inhibitors reversed the significant tumorigenic difference between the injection of IL-23 (50 ng) and vehicle groups that comprised of IL-23R^+^ TE-1 cells. **, *p* <0.01.


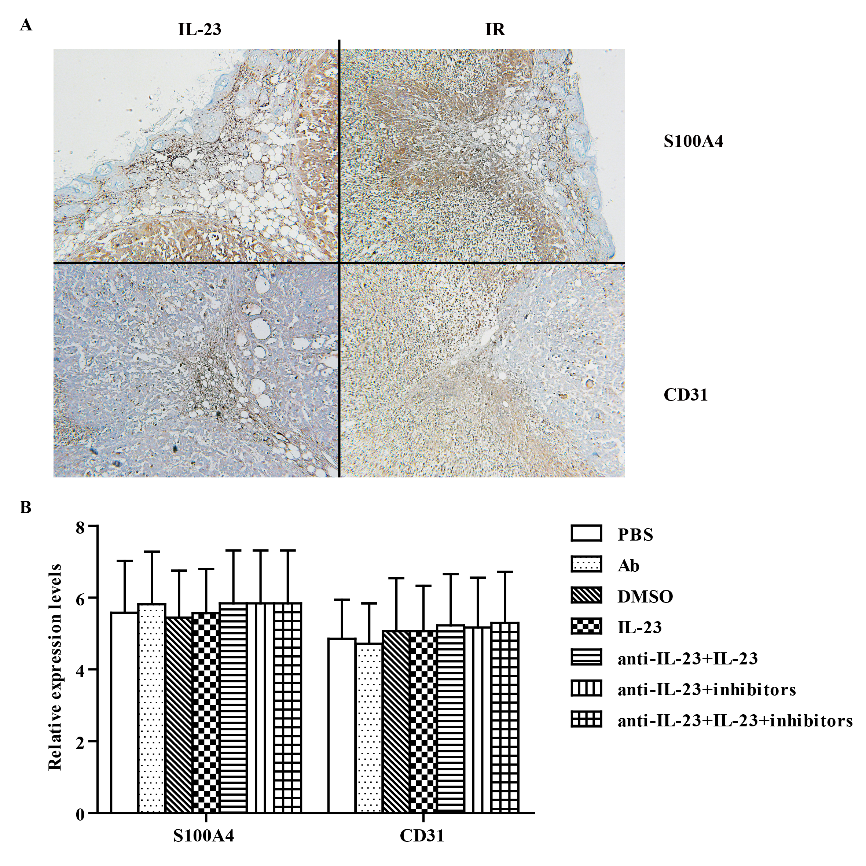


Figure S4. The fibroblast and endothelial cells in ESCC xenograft models. A. Representative figures of fibroblast and endothelial cells in tissues sections from xenograft model with ESCC (left panel: IL-23 treatment, right panel: irradiation treatment). B. The expression change of fibroblast and endothelial cells makers (anti-mouse antibodies, S100A4 and CD31) with indicated treatments were measured by IHC. A, B. 200× magnification.
