# Supplementary material for: Interleukin-23 receptor signaling mediates cancer dormancy and radioresistance in human esophageal squamous carcinoma cells via the Wnt/Notch pathway
Source: J Mol Med (Berl). 2018 Nov 27;97(2):177–88. doi: 10.1007/s00109-018-1724-8 (PMC6348073; doi:10.1007/s00109-018-1724-8)
Supplement: Supplementary file 2 — (DOC 57 kb) [file 109_2018_1724_MOESM2_ESM.doc]

| **Supplementary Tabel 1. The correlation of IL-23 expression and clinical-pathological features in ESCC patients** | | | | | |
| --- | --- | --- | --- | --- | --- |
| **Clinical-pathological Features** | **Number** | **IL-23 (tumor)** | | **IL-23 (para-carcinoma)** | |
|  | (n=56) | (Mean ± SD) | *p* value | (Mean ± SD) | *p* value |
| **Gender** |  |  |  |  |  |
| Male | 29 | 11.17 ± 5.88 | 0.451 | 5.14 ± 0.64 | 0.314 |
| Female | 27 | 10.04 ± 5.27 |  | 4.96 ± 0.65 |  |
| **Age** |  |  |  |  |  |
| ≤50 | 16 | 10.13 ± 5.25 | 0.675 | 5.19 ± 0.54 | 0.33 |
| >50 | 40 | 10.83 ± 5.75 |  | 5.00 ± 0.68 |  |
| **Tumour location** |  |  |  |  |  |
| Upper/Middle | 30 | 10.00 ± 5.62 | 0.372 | 5.27 ± 0.58 | 0.07 |
| Lower | 26 | 11.35 ± 5.53 |  | 4.89 ± 0.63 |  |
| **Differentiation level** |  |  |  |  |  |
| Well differentiated | 19 | 10.05 ± 4.40 | 0.857 | 5.00 ± 0.58 | 0.822 |
| Moderately differentiated | 21 | 10.83 ± 6.29 |  | 5.04 ± 0.71 |  |
| Poorly differentiated | 16 | 11.07 ± 6.06 |  | 5.14±0.66 |  |
| **Lymphatic metastasis** |  |  |  |  |  |
| No | 25 | 5.48 ± 2.12 | **0.000** | 5.04 ± 0.54 | 0.889 |
| Yes | 31 | 14.77 ± 3.67 |  | 5.06 ± 0.73 |  |
| **Distant metastasis** |  |  |  |  |  |
| No | 48 | 9.33 ± 4.87 | **0.010** | 5.02 ± 0.64 | 0.356 |
| Yes | 8 | 18.38 ± 2.20 |  | 5.25 ± 0.71 |  |
